# A Case Report of Primary Mediastinal Liposarcoma: A Rare Mediastinal Tumor

**DOI:** 10.7759/cureus.66293

**Published:** 2024-08-06

**Authors:** Ghaidaa A Almuhammadi, Rawia A Alzughaibi, Raha Z Ishqi, Mervat Aboualkheir, Amr M Allama

**Affiliations:** 1 Collage of Medicine, Taibah University, Madinah, SAU; 2 Department of Radiology and Medical Imaging, Taibah University, Madinah, SAU; 3 Department of Thoracic Surgery, King Fahad General Hospital, Madinah, SAU

**Keywords:** sarcomas, tumor, mediastinal, myxoid, liposarcoma

## Abstract

Liposarcomas account for about 20% of all sarcomas among mesenchymal neoplasms. Myxomatous liposarcoma is a rare mediastinal tumor that seems the same as other lung disorders. The most common presenting symptoms are chest pain, dyspnea, and dysphagia. Most of the diagnostic findings are provided by radiological or postoperative histopathological tests. Surgery and chemotherapy, in some cases, are the basis of treatment. People with this condition have a higher probability of a favorable outcome if they receive an early diagnosis and treatment. We present a case of a 48-year-old male with primary mediastinal liposarcoma. The patient complained of chest pain and shortness of breath with a productive cough. Computed tomography (CT) showed a large right cystic mass on the right lower thoracic cavity. Surgery was done, and a histopathological examination of the surgical specimen confirmed the diagnosis.

## Introduction

Liposarcoma in the mediastinum is relatively uncommon, accounting for about 1% of all cases. Although it mainly affects the posterior mediastinum [1], primary liposarcoma of the mediastinum has a rate of 1.6%-2.5% [2]. However, the most common sites where the tumor is usually found are the retroperitoneum and lower limbs [1]. Based on histopathological characteristics, liposarcoma is classified into the following five subtypes: well-differentiated, mucous, dedifferentiated, pleomorphic, and myxoid/round cell [3]. The subtype with the highest incidence is well-differentiated liposarcoma, followed by myxoid liposarcoma. It makes up approximately 15%-25% of all cases of liposarcomas and constitutes 5% of all adult soft tissue tumors [4].

## Case presentation

**Figure 1:**
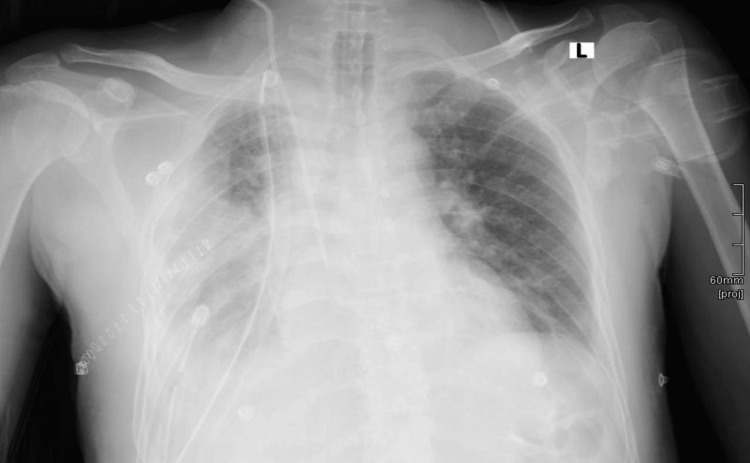
Chest X-ray showed a massive pleural effusion on the right side.

We report a case of a 48-year-old male who was medically free. He complained of right-side chest pain and shortness of breath with a productive cough for the past month with a gradual onset and progressive course. There was no weight loss, night sweating, loss of appetite, or dysphagia. Physical examination revealed dullness on percussion and decreased breath sounds on the right side. A chest X-ray showed a massive pleural effusion on the right side (Figure 1).

**Figure 2:**
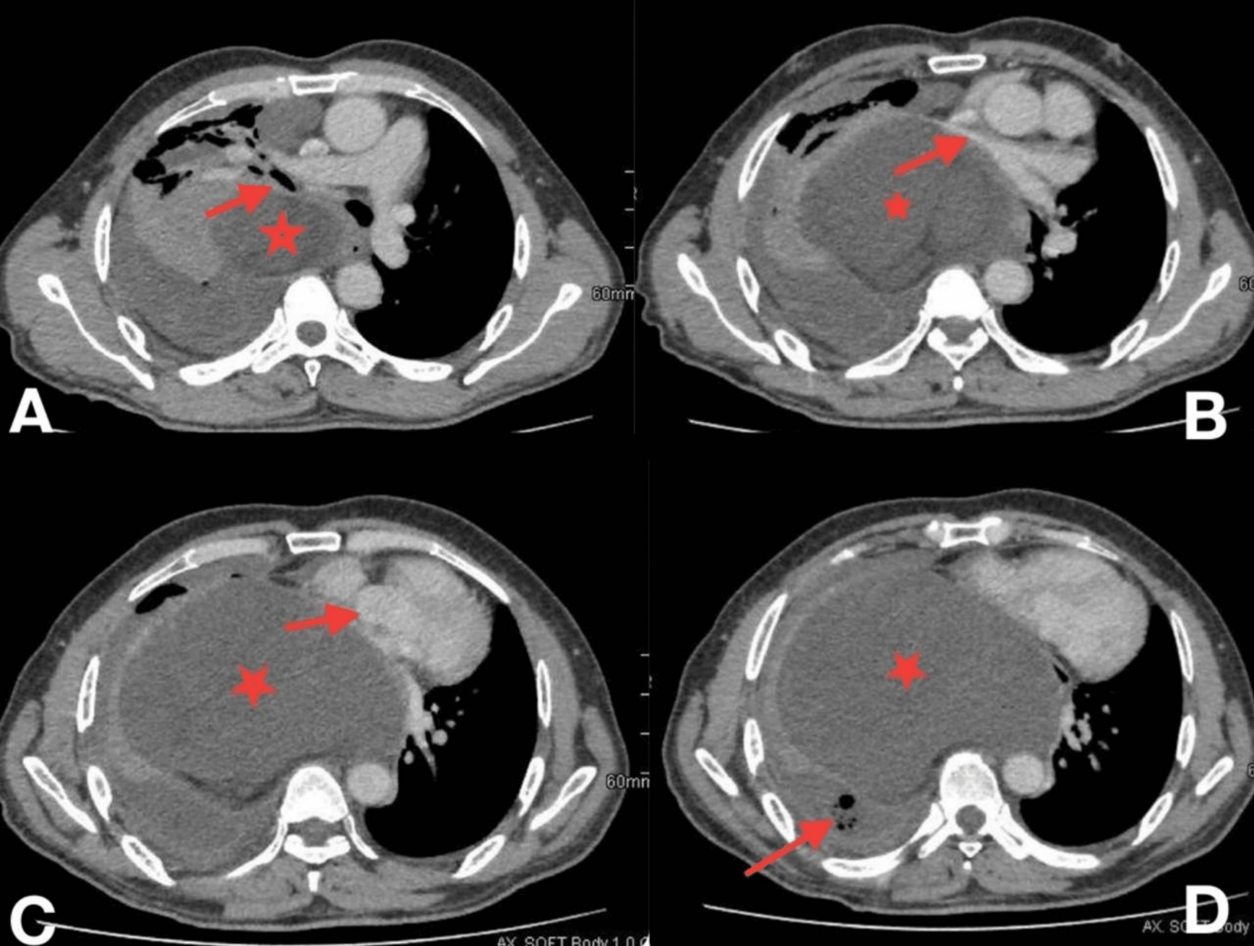
Enhanced axial chest CT scan soft tissue window shows a large mass (star) extended to the mediastinum causing compression to the right main bronchus (arrow) (A), with a significant displacement of the cardiovascular mediastinal structures toward the left side with an attenuation of the lumen of pulmonary veins (arrow) (B), left and right atrium (arrow) (C), and multiloculated right pleural effusion with thick irregular pleural enhancement and multiple septations associated with gas bubbles (arrow) (abscess) (D). CT: computed tomography

**Figure 3:**
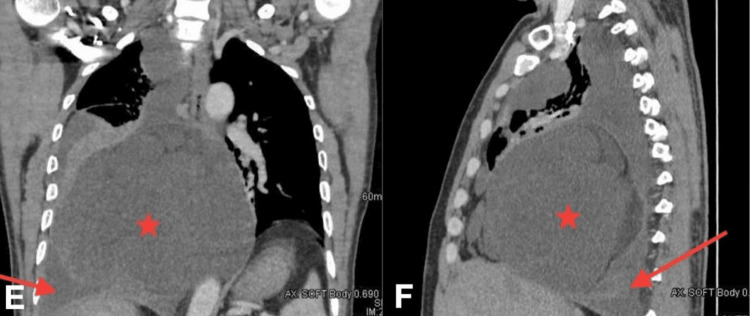
Enhanced coronal (E) and sagittal (F) chest CT scan soft tissue window shows a large well-demarcated mass (star) with peripheral enhancement with the main bulk of it in the right lower thoracic cavity and to a lesser extent in the mediastinum and multiloculated right pleural effusion with thick irregular pleural enhancement and multiple septations (arrow). CT: computed tomography

Furthermore, an enhanced computed tomography (CT) scan reveals a multiloculated right pleural effusion with thick irregular pleural enhancement, multiple septations, and gas bubbles, which raise concerns about its chronicity and the potential formation of an abscess. Additionally, lymphadenopathy is evident, with a 5 mm right internal mammary lymph node and a 7 mm right epiphrenic lymph node observed. Fortunately, the upper abdominal sections of the scan appear unremarkable, and no aggressive bone lesions are noted in the evaluation (Figure 2 and Figure 3).

**Figure 4:**
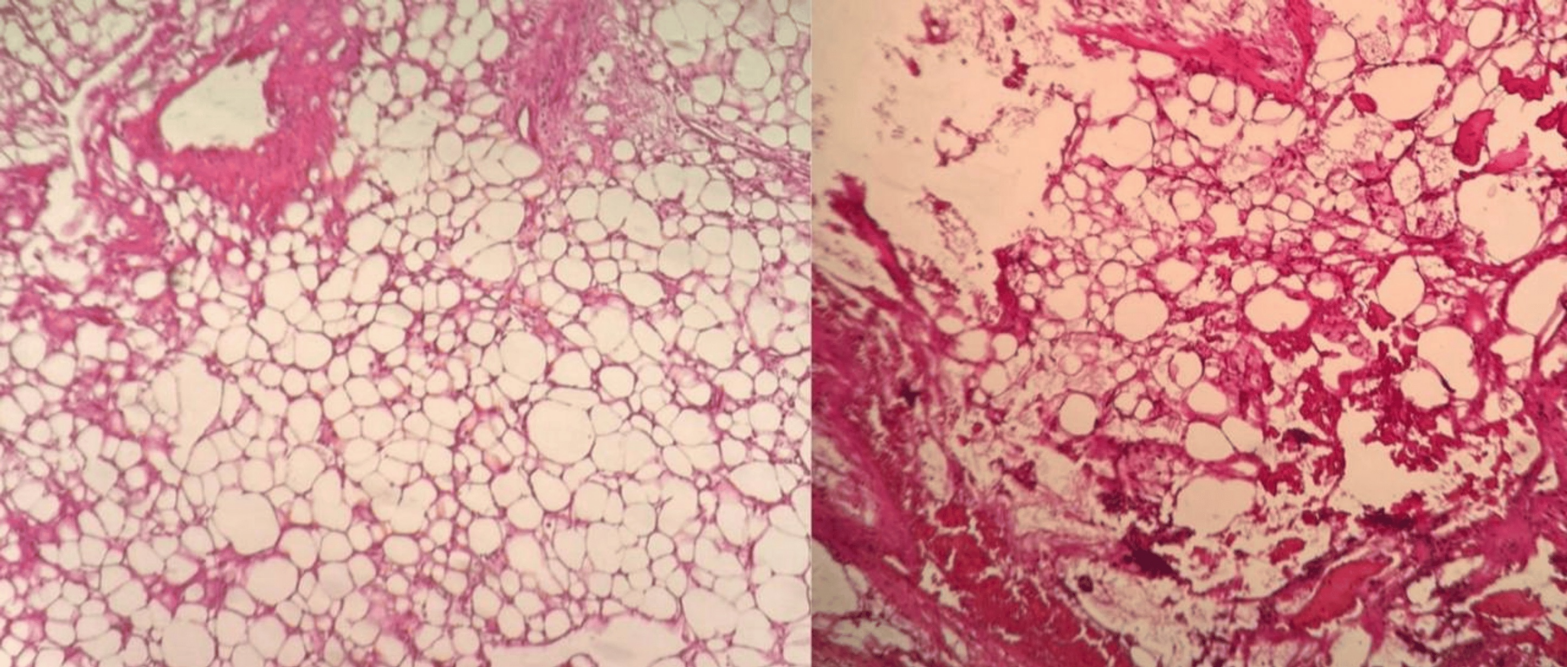
Postoperative H&E: ×100.

A pleural tap was performed on the day of the admission, and about 400 mL of hemorrhagic fluid was collected from the right side. The patient underwent surgery after two weeks of admission, a right-sided thoracotomy with a right-side chest tube inserted. Postoperatively, the patient was admitted to the intensive care unit (ICU) for eight days and was on mechanical ventilation for two days. A postoperative histopathological examination showed soft tissue yellow in color and myxoid inconsistent with abundant mucin measures (14×9 cm). Sections show tumors composed of lobules of monomorphic, stellate, or fusiform-shaped cells in a myxoid background with irregular cystic spaces. Delicate thin-walled arborizing and curving capillaries are present. Numerous lipoblasts are present in the periphery of the lobules, with no significant mitotic activity (Figure 4).

Differential diagnosis includes myxoid liposarcoma and well-differentiated liposarcoma with abundant myxoid stroma. Mouse double minute 2 (MDM2) immunohistochemical stain and fluorescence in situ hybridization (FISH) testing were not performed due to unavailability. After three weeks, the patient was symptom-free, discharged, and arranged to follow up in the clinic.

## Discussion

Liposarcoma is a soft tissue cancer that develops from mesenchymal tissues. It can occur in any body region but is most common in the retroperitoneum and soft tissue of the limb trunk [5]. It is responsible for 20% of all sarcomas found in the body [1].

The median age at diagnosis was 43-49 years old [6]. Eighty-five percent of patients diagnosed with liposarcoma show symptoms, while the remaining 15% have no recognized or distinguishing signs. A report presents several cases that were accidentally discovered [7]. In the early stages, there were no obvious clinical symptoms; as the tumor size, location, and pressure or the invasion of surrounding tissues increased, it resulted in pain, edema, and other symptoms such as shortness of breath, chest pain, wheezing, hoarseness, superior vena cava compression, arrhythmias, and heart failure caused by intrathoracic structure compression [8].

The tumor is distinguished by increased expression in the cyclin-dependent kinase 2 (*CDK2*) and *MDM2* genes on chromosome 12 [9]. In addition to elderly age without consideration for gender, exposure to radiation and harmful substances has been recognized as a significant risk factor for the disease [10].

The diagnosis of liposarcoma is mainly based on imaging findings such as chest X-rays, computed tomography (CT), magnetic resonance imaging (MRI), and histopathological examination. On chest radiography, there is a chance that tracheal deviation will be visible. Fine-needle aspiration (FNA) can also be an additional diagnostic tool [11,12]. Using CT and MRI, it is feasible to see a fatty mass containing other soft tissue components. However, preoperative radiological liposarcoma and lipoma diagnosis are challenging; histopathological diagnosis and typing are essential [12,13]. The diagnosis can be confirmed with fine-needle biopsy and postoperative pathological biopsy [13].

The histological subtype of a malignancy plays a crucial role when assessing the disease's prognosis. Tumors that have been well-differentiated or undifferentiated are the least aggressive. Both myxoid and pleomorphic subtypes are aggressive; they may migrate and proliferate on the pleural, pericardial, and diaphragmatic surfaces of the body. Furthermore, these tumors have a high recurrence rate [14]. Old age at diagnosis, tumor size, grade, and depth, as well as the presence or absence of tumor-free margins, are all factors that influence the prognosis of myxoid liposarcoma [15].

Liposarcomas have been treated mainly by surgical excision. Small mediastinal liposarcomas can be removed using a minimally invasive technique that includes a video-assisted or machine-assisted thoracoscope, whereas larger tumors frequently need open surgery; the median sternotomy or lateral thoracotomy has the best chance of success and is most commonly used for the complete excision of the mediastinal tumor [13,16]. Chemotherapy and radiation therapy are unlikely to be efficient in treating this tumor. CDK2 inhibitors can treat patients whose tumors were only partially excised [17].

## Conclusions

Myxomatous liposarcoma is a rare mediastinal tumor with characteristics like other lung conditions. Mediastinal liposarcomas vary in morphology; they can be aggressive and lethal. The diagnoses are based on radiological findings and postoperative histopathological examination. The treatment is mainly surgery. Early detection and treatment can lead to a better outcome. Therefore, a close follow-up is strongly suggested.
